# Surgery

**Published:** 1904-09

**Authors:** T. Carwardine, C. A. Morton


### SURGERY.

**The Surgical Treatment of Bright's Disease.** — Sufficient time has now elapsed since the introduction of nephropexy as a cure for Bright's disease to form a more accurate opinion of its real value. We have already referred to the original paper by Edebohls,[2] published two years ago, giving the details of his technique in operations for movable kidney. From that paper we quote the following extract:—"No less than sixteen of my own cases of nephropexy came to operation with chronic nephritis, and one with acute nephritis. Not only was the inflammation of the kidneys not aggravated in any case, but the great majority of the patients were permanently cured of their nephritis as one result of the operation. Six of the patients suffering with nephritis at the time nephropexy was performed upon them are in perfect health, and the urine remains free from albumin and casts at periods varying from two to over eight years after operation." Now this was perhaps the view of an enthusiast, but we are now able to apply the experience of others to a correct estimation of its value ; and, in passing, we must give our American friends the full credit for prosecuting this branch of surgical inquiry.

The subject was somewhat fully discussed by the Chicago Surgical Society recently,[3] and it may be profitable to consider the various views expressed; and in particular those of Dr. Emil Ries, which are opposed to the opinions as to the pathological causes of operative success. Dr. Ries had a patient, aged 44, with hæmaturia from the left kidney, the urine from the right kidney being normal. In August, 1901, left nephropexy was performed, but the hæmaturia returned after a

[1] *Dublin J. M. Sc.*, 1903, cxiv., No. 384. [2] *Ann. Surg.*, 1902, xxxv. 169.

[3] *Ibid.*, 1904, xxxix. 617.

See also *Bristol M.-Chir. J.*, 1902, xx. 241, 336; 1904, xxii. 62.

short interval. A few months after the first operation the left kidney was removed. The newly-formed capsule around the kidney was about as thick as one's little finger, and was very firmly attached all round, so that it would have been difficult to remove the kidney with the capsule; but it was easy to detach the capsule from the kidney itself and remove the kidney. From the kidney removed over 600 sections were made and examined, and among the 600 sections there was but one single anastomosis, and this in the path of an incision into the pelvis of the kidney, merely a vessel in scar tissue.

This experiment in the human subject, if it may be so called, proved conclusively that the theory of the *modus operandi* of this operation, to wit, that it was due to a newly-formed anastomosis, was incorrect. But the incorrectness of the theory did not necessarily prove the incorrectness of the treatment. From his experience in five cases he was sceptical as to the results of this operation, and had two fatal cases to record, *i.e.* fatal within seven months of operation. Operation did not improve the prognosis in those cases in which there was disturbance in the fundus oculi. In cases of floating kidney, with the presence of albumin in the urine, improvement seemed to follow operation with decapsulation to a greater extent than without it; but as a treatment for chronic interstitial nephritis, he would never advise the operation, although he would operate as an experiment if asked by the patient to do so.

Dr. Arthur Dean Bevan did not think renal decapsulation could be called the surgical treatment of Bright's disease, because, as a fact, the great majority of surgeons throughout the United States and of the world had never accepted the operation as being a logical procedure. . . . Stripping off the capsule for the cure of Bright's disease was of little, if any, value; and it now only remained for the profession to convince Ferguson, Edebohls, and others of this fact. Dr. E. W. Andrews reported four cases. In three there was no improvement; in the fourth, he thought it was likely that the nephropexy and not the decortication did the work of relief from symptoms of intermittent hydronephrosis with unilateral nephritis. . . . Dr. L. L. McArthur gave the following conclusions from a case of double decapsulation of the kidney: General improvement, improvement in the urine from the first kidney operated on, improvement in that from both kidneys after the second kidney was operated on. Dr. Ochsner had operated on seven cases, all but one of which seemed hopeless, and in all but one there was marked albuminuric retinitis. Four out of the seven had died within one year after the operation, one of them within a few weeks. One improved materially after operation on one kidney, and expressed the desire to have the other kidney operated on; but he died in seven days from acute uræmia. Three were alive, including one operated on only a few weeks previously. Dr. A. H. Ferguson said that in the milder cases of nephritis the results of decapsulation were beautiful, both immediate and permanent. He regarded the mortality of renal decapsulation *per se* as about 6 per cent.; but that, after operation, many cases died from the original disease, not merely from surgical intervention. He had years ago refused to operate on cases of floating kidneys because there was Bright's disease as well. Now it was an indication, the indication being the nephritis itself. He did not mention his actual mortality from operation, but of the five cases of which he gave details, two died and three were improved.

From a consideration of the cases described, one is led to the conclusions, firstly, that many cases of mechanical nephritis from wandering kidney have been included in the reports; secondly, that, although in some cases wonderful improvement has taken place, yet the mortality within a comparatively short time of operation will prevent judicious surgeons from adopting decapsulation as a certain cure for Bright's disease. And one of the strongest advocates for the procedure, after operating on a case with general œdema, said, "Nothing surgically could be done which would be of benefit in these cases of small white kidney." The sooner surgeons cease to operate on such cases, therefore, the better for the reputation of the art they practise. Nor must we forget that the strongest advocate of all is he who fixes the decapsulated kidney, removes the appendix, and fixes the uterus all at one sitting—a sense of operative proportion which scarcely appeals to most surgeons; and which, perhaps, deserves the designation of Dr. A. D. Bevan as the gynæcological treatment of Bright's disease!

T. CARWARDINE.

The visit of Lorenz to this country and his demonstration of his method of treating **congenital dislocation of the hip-joint** by manipulation and fixation in plaster[1]—"the bloodless method" as it has been called, to distinguish it from the open operation —has called the attention of surgeons to the treatment of this deformity. An interesting paper on the subject was read by Mr. Burghard[2] in the section of Children's Diseases at the last British Medical Association meeting, and various other papers and letters have been published.[3] It is generally agreed that the Lorenz bloodless method is the best one to employ, and that an operation should only be performed if it fails. By manipulation under an anæsthetic the head is reduced so as to lie against the imperfect acetabulum, and it is fixed thus by encasing the limb and pelvis in plaster for many months. Lorenz believes that in a large number of the cases it remains there, but that in those in which it does not it lies in a position

[1] *Brit. M. J.*, 1903, i. 163. [2] *Ibid.*, 1903, ii. 457.
[3] *Ibid.*, 1903, i. 137, 225, 373.

which enables the patient to walk about much better than he did before. That the method usually leads to considerable improvement in the patient's walk most surgeons who have tried the method admit, but many consider that only very exceptionally does the head remain at the acetabulum. Mr. Muirhead Little[1] and Mr. Burghard[2] have shown by statistics of the results obtained by various continental surgeons how frequently reluxation occurs, and Mr. Burghard states that out of thirty cases of his own, in only one was there a true permanent reduction, though the gait of a great many was much improved. This fact should make us careful to follow up cases for a considerable period before judging of the results of the method. Mr. Burghard says, in speaking of the results of the bloodless method, that great improvement follows in the majority of cases provided that the patient is of a suitable age, but that there is little reliable information at present as to whether the false joint formed is permanently stable. The suitable age as given by Lorenz is up to the ninth to tenth year for unilateral cases and the seventh to eighth for bilateral.[3] Mr. Burghard himself is in favour of operation by his own method after five years of age, rather than non-operative manipulation. Bilateral cases are found to be, as one might expect, more difficult to treat than unilateral ones. In Lorenz's bloodless manipulation, when the child is under the anæsthetic, by a series of sharp blows with the ulnar border of the hand he subcutaneously breaks away the attachment of the adductor to the pelvis. Then by traction downwards with extreme abduction the head is brought down to and forced against what there is of an acetabular cavity, and it is put up in plaster in a position of extension and extreme abduction, and allowed to remain so for several months. The exact number of months varies in the different published accounts of the method, but in the abstract of Lorenz's paper[4] it is stated to be four to five months without walking, and then four to five months more with walking on the limb. In order to allow such walking Mr. Burghard[5] states the degree of abduction is reduced and slight flexion substituted for complete extension. During walking the head is pressed against the imperfect acetabulum and deepens it.

In the Hoffa-Lorenz operation as practised at the present time a new acetabular cavity is gouged out of the pelvic bone, and the head is if necessary trimmed to fit it. The chief risk of such an operation is the occurrence of anchylosis. The risks of the operation as regards the life of the patient are not great. Hoffa had seven deaths in 248 cases from sepsis, but no death in his last series of 132 cases. Mr. Burghard[6] and Mr. Tubby[7] replace the head of the bone in the acetabulum by open incision, without deepening the acetabular cavity, and Mr.

[1] *Brit. M. J.*, 1903, i. 225. [2] *Ibid.*, 1903, ii. 461.
[3] *Lancet*, 1900, ii. 830. [4] *Loc. cit.* [5] *Loc. cit.*, 460. [6] *Loc. cit.*, 459.
[7] *Brit. M. J.*, 1903, ii. 463.

Burghard advocates this method in cases in which manipulation fails to give marked improvement and in children over five years of age. He points out that the bone can be thus placed with absolute certainty in the acetabulum after opening up the contracted part of the capsule, which often lies over its lower end, and that the tendency for it to slip out again can be partly corrected by removing and suturing the upper lax part of the capsule. Before undertaking this operation Mr. Burghard divides the adductors, but Mr. Tubby removes a portion of them, and of the sartorius and tensor fasciæ femoris, and puts the limb in plaster in full abduction and extension for six weeks before he operates on the joint.

I have referred to the risks of the Hoffa-Lorenz operation. Mr. Burghard has had no mortality in the thirteen cases operated on by his own method, but Lorenz's bloodless method is not wholly devoid of risk. Lorenz has had in 450 cases eleven fractures of the neck of the femur, two of the pelvis, three of permanent paralysis of the sciatic nerve, as well as temporary paralysis of the nerves,[1] and Mr. Robert Jones in four cases out of thirty-eight fractured the neck of the femur with transposition of the shafts anteriorly.[2]

C. A. Morton.

### Reviews of Books.

[1] *Brit. M. J.*, 1903, i. 225. [2] *Ibid.*, 1903, ii. 463.

